# A genomic island in *Vibrio cholerae* with VPI-1 site-specific recombination characteristics contains CRISPR-Cas and type VI secretion modules

**DOI:** 10.1038/srep36891

**Published:** 2016-11-15

**Authors:** Maurizio Labbate, Fabini D. Orata, Nicola K. Petty, Nathasha D. Jayatilleke, William L. King, Paul C. Kirchberger, Chris Allen, Gulay Mann, Ankur Mutreja, Nicholas R. Thomson, Yan Boucher, Ian G. Charles

**Affiliations:** 1 University of Technology Sydney, School of Life Sciences, Sydney, 2007, Australia; 2 University of Alberta, Department of Biological Sciences, Edmonton, T6G 2E9, Canada; 3 University of Technology Sydney, The ithree institute, Sydney, 2007, Australia; 4 Defence Science and Technology Group, Melbourne, 3207, Australia; 5 Wellcome Trust Sanger Institute, Wellcome Trust Genome Campus, Hinxton, CB10 1SA, United Kingdom

## Abstract

Cholera is a devastating diarrhoeal disease caused by certain strains of serogroup O1/O139 *Vibrio cholerae*. Mobile genetic elements such as genomic islands (GIs) have been pivotal in the evolution of O1/O139 *V. cholerae*. Perhaps the most important GI involved in cholera disease is the *V. cholerae* pathogenicity island 1 (VPI-1). This GI contains the toxin-coregulated pilus (TCP) gene cluster that is necessary for colonization of the human intestine as well as being the receptor for infection by the cholera-toxin bearing CTX phage. In this study, we report a GI (designated GI*Vch*S12) from a non-O1/O139 strain of *V. cholerae* that is present in the same chromosomal location as VPI-1, contains an integrase gene with 94% nucleotide and 100% protein identity to the VPI-1 integrase, and attachment (*att*) sites 100% identical to those found in VPI-1. However, instead of TCP and the other accessory genes present in VPI-1, GI*Vch*S12 contains a CRISPR-Cas element and a type VI secretion system (T6SS). GIs similar to GI*Vch*S12 were identified in other *V. cholerae* genomes, also containing CRISPR-Cas elements and/or T6SS’s. This study highlights the diversity of GIs circulating in natural *V. cholerae* populations and identifies GIs with VPI-1 recombination characteristics as a propagator of CRISPR-Cas and T6SS modules.

*Vibrio cholerae* is a species of bacteria commonly found in marine and estuarine waters and the causative agent of the diarrhoeal disease cholera^1^. Although more than 200 serogroups of the bacterium are known, only two, O1 and O139, are responsible for pandemics of the cholera disease^2^. Historically, there have been seven pandemics of cholera, with the current seventh pandemic caused by O1 strains of the El Tor biotype and the sixth and presumably the previous five pandemics caused by O1 strains of the classical biotype^1^. The evolution of pandemic strains has been greatly influenced by lateral gene transfer (LGT), which led to the acquisition of many novel virulence factors^3^.

Integrative mobile genetic elements (MGEs) such as transposons and genomic islands (GIs) are particularly important in LGT processes, as they help facilitate integration of non-homologous transferred DNA into replicons such as the chromosome or a resident plasmid(s)^4^. Studies comparing the genomes of *V. cholerae* strains consistently find MGEs as one of the sources, if not the major source, of genome variation^5^,^6^. For example, the seventh pandemic strains contain two GIs called the *Vibrio* seventh pandemic islands I and II not present in the strains isolated from the previous pandemics^7^. Most important to pathogenicity of the pandemic strains are genes found on two MGEs, the cholera toxin bacteriophage (CTXϕ) and a GI called the *V. cholerae* pathogenicity island 1 (VPI-1)^8^,^9^. Although these MGEs are common in pandemic strains, examples of CTXϕ and VPI-1 in non-O1/O139 strains have been documented and are indicative of their mobility across natural populations of *V. cholerae* and close relatives^10^,^11^,^12^. The CTXϕ contains genes encoding a potent enterotoxin that, when secreted in the human intestinal tract, results in significant loss of fluid that can lead to death within 24 hours if left untreated^1^. VPI-1 contains the genes encoding the toxin-coregulated pilus (TCP; thus, VPI-1 is also known as the TCP pathogenicity island) that is required for adhesion to the intestinal wall as well as genes encoding the accessory colonization factor and the regulatory genes *toxT* and *tcpPH*^13^,^14^,^15^. TCP is also the receptor for CTXϕ and thus a prerequisite for infection and subsequent lysogeny in the emergence of toxigenic *V. cholerae*^8^.

Another virulence factor associated with frequent LGT is the type VI secretion system (T6SS) of *V. cholerae* and other Gram-negative bacteria. Bacteria that harbor T6SS produce a membrane-spanning protein complex capable of puncturing eukaryotic or prokaryotic cells and injecting toxic effector proteins into their targets^16^. In *V. cholerae*, the presence of T6SS has previously only been reported as a conserved chromosomal element and displays toxic activity against macrophages^17^. The T6SS is made up of a series of proteins encoded by genes in three different locations of the *V. cholerae* genome: a main cluster on chromosome 2 and two smaller auxiliary clusters on chromosomes 1 and 2^17^,^18^. The characteristic proteins of the T6SS are Hcp and VgrG. Hcp is encoded by two alleles on the *V. cholerae* chromosome and polymerizes to form the nanotube that protrudes from the bacterial cell surface^19^,^20^. The tip of Hcp is decorated with VgrG proteins that form a trimer^21^. VgrG proteins are conserved at their N-termini but carry specialist C-termini with enzymatic activity^21^. For example, *V. cholerae* contains three VgrG alleles with two encoding proteins with actin cross-linking (VgrG-1) and peptidoglycan degrading (VgrG-3) activity^22^,^23^. Additional effector proteins with diverse enzymatic activities can be added to the VgrG spike for delivery into target cells^24^.

Acquisition of novel genes through the uptake of MGEs can have obvious beneficial effects for harmless and pathogenic bacteria alike. However, not all LGT is advantageous, and bacteria have evolved a variety of methods to prevent the spread of harmful DNA sequences in their genomes^25^. One recently discovered defense mechanism against unwanted LGT is the CRISPR-Cas system. CRISPR-Cas modules consist of *cas* genes and an array of short direct repeats separated by highly variable spacer sequences that correspond to genetic elements such as bacteriophages or MGEs^26^. Transcription of the CRISPR array with subsequent slicing of the transcript into smaller CRISPR RNAs acts in concert with the Cas proteins to specifically recognize foreign DNA and cleave it^26^. The system acts as an adaptive immune system for bacteria as it allows for the synthesis and incorporation of new spacers into the array following invasion of a foreign DNA molecule, thus providing immunity to the host cell.

Non-O1/O139 strains of *V. cholerae* have been hypothesized to be an important source of new MGEs that could relocate into pandemic strains. Here, we report a GI with VPI-1 recombination characteristics that harbors both a CRISPR-Cas module and an auxiliary T6SS locus in a non-O1/O139 strain of *V. cholerae* from Sydney, Australia. This GI likely provides recipient cells not only with a defense mechanism against maladaptive LGT, but also with a potential competitive advantage over bacteria lacking this GI and perhaps a novel virulence factor. We also show that similar GIs are present in other non-O1/O139 strains from around the globe.

## Methods

### Bacterial strain, growth conditions, and molecular biology methods

The non-O1/O139 *V. cholerae* S12 strain was isolated from the Georges River (Sydney, Australia) in 2009^27^ and routinely cultured on Luria-Bertani medium at 37 °C. The whole genome of *V. cholerae* S12 was paired-end sequenced at the Wellcome Trust Sanger Institute (Hinxton, UK) using Illumina HiSeq 2000 (San Diego, CA, USA) from DNA extracted using the Wizard Genomic DNA Purification Kit (Promega, Madison, WI, USA). For extraction of GI*Vch*S12 circles, a plasmid extraction was carried out on S12 using the PureYield Plasmid Miniprep Kit (Promega). PCR was performed using 2X MangoMix (Bioline, London, UK) that consists of DNA polymerase, dNTPs, Mg^2 + ^and an orange dye with 30 cycles of denaturation at 93 °C for 30 sec, the appropriate annealing temperature for 30 sec and an extension of 72 °C (1 min/kb). All primers were acquired from Integrated DNA Technologies (Coralville, IA, USA) (Table 1) and used at a final concentration of 10 μM. PCR amplicons intended for sequencing were excised from 1% agarose gels and purified using the Wizard SV Gel and PCR Clean-Up Kit (Promega) and sequenced using the Sanger method at Macrogen (Seoul, South Korea).

### Bioinformatic analyses

The Illumina HiSeq whole genome sequencing reads for *V. cholerae* S12 were filtered to remove low quality reads with average read quality less than Q20 and low quality trailing ends with base quality less than Q20 using Prinseq-lite v0.20.4^28^. Reads were then *de novo* assembled into contiguous sequences (contigs) using Velvet v1.2.10^29^ and the assemblies were improved by scaffolding using SSPACE v2.0^30^, gap filling using GapFiller v1.10^31^, reordering of contigs against the *V. cholerae* N16961 reference genome using Mauve v2.4.0^32^ and removal of contigs shorter than 300 bp. The final improved draft genome assembled into 83 scaffolds from 4,624,354 read pairs with an average read length of 75 bp to give a total genome size of 4,061,577 bp with average depth of coverage of 171 reads.

**Figure 1:**
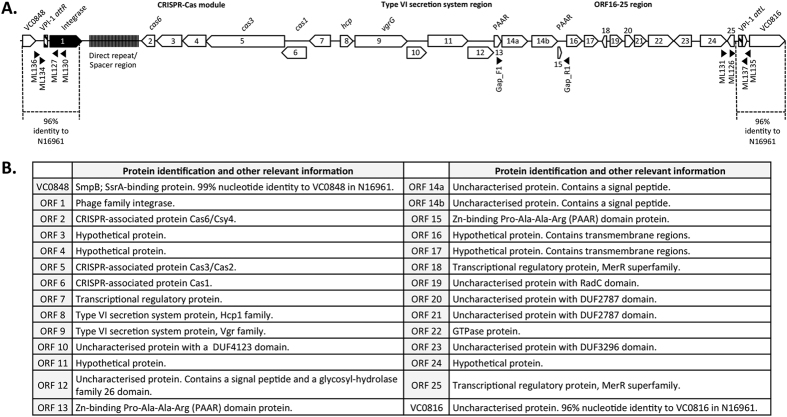
The ~28-kb genomic island, GI*Vch*S12, from *Vibrio cholerae* S12 containing a CRISPR-Cas module and type VI secretion auxiliary locus. Schematic representation of GI*Vch*S12 is shown in (**A**) with the VPI-1 *att* sites given as hatched boxes and the VPI-1 integrase as a black block arrow. Regions of nucleotide identity to VPI-1 and surrounding regions in *V. cholerae* N16961 are shown. All ORFs and their orientation are shown as block arrows. Numbers shown in, above, or below the block arrows correlate to the putative identification shown in (**B**). Primer binding sites are shown by black triangles with their direction of extension indicated by the direction of the triangle.

The genomic region encoding GI*Vch*S12 was identified on contig 000009 by pairwise comparison of the *V. cholerae* S12 draft genome to the complete reference genome of *V. cholerae* N16961 using the program Mauve v2.4.0^32^. Analysis of GI*Vch*S12 on contig 000009 identified three assembly gaps between ORFs 13 and 15 due to a putative repeat of ORF14 (annotated as ORF14a and ORF14b). To confirm this repeat, a PCR with primers Gap_F1 and Gap_R1 that anneal outside of this repeat region (see Fig. 1) resulted in an expected ~2.5 kb product (as opposed to a ~1.3–1.5 kb product if only one copy of ORF14 was present). Two of the assembly gaps were closed through sequencing the ends of the amplicon creating the GI*Vch*S12 sequence in GenBank file KU722393. GI*Vch*S12 was annotated using Prokka^33^ and the automated annotation was manually curated with the aid of BLAST against the non-redundant NCBI protein database (Bethesda, MD, USA) using BLASTP providing putative identification^34^. The CRISPR-Cas module in GI*Vch*S12 was identified using the online tool CRISPRFinder (http://crispr.i2bc.paris-saclay.fr)^35^.

In order to determine if GI*Vch*S12 or similar islands were present in other *V. cholerae*, additional *V. cholerae* genomes were obtained from GenBank. The list of genomes used is provided in Supplementary Material. The genomes were annotated with RAST v2.0 (Rapid Annotation Using Subsystem Technology)^36^. The GI*Vch*S12 ORFs were compared against the ORFs of the *V. cholerae* genomes to determine presence/absence by calculating the BLAST score ratio (BSR)^37^. Significant hits were considered as putative homologues if the BSR values were at least 0.3 (for 30% amino acid identity)^38^. Furthermore, the whole genomes were aligned using Mugsy v1.2.3^39^, and the core alignment (2,539,853 bp in total length) was extracted from the Mugsy output using Galaxy v16.04^40^ and Geneious v8.1.8^41^. From this alignment, a maximum likelihood phylogenetic tree was constructed using RAxML v8.1.17^42^ using the general time reversible (GTR) nucleotide substitution model and gamma distribution pattern. Robustness of branching was estimated with 100 bootstrap replicates. *Vibrio metoecus*, the closest relative of *V. cholerae,* was used as an outgroup^43^.

### GenBank accession numbers

The full sequence of the GI*Vch*S12 including flanking sequences and the sequenced *attP* and *attB* sites are available in GenBank/ENA/DDBJ and have the accession numbers KU722393, KU722394, and KU722395, respectively. The raw Illumina HiSeq sequencing reads are available under accession number ERR063652 and the improved draft genome assembly of *V. cholerae* S12 can be accessed at MDST00000000.

## Results and Discussion

### A novel variant of the *Vibrio* pathogenicity island

In order to identify regions of interest in the genome of *V. cholerae* S12, contigs were compared to the closed genome of *V. cholerae* N16961. A novel GI in the same respective location as VPI-1 (between VC0816 and VC0848 on chromosome 1) was identified on contig 000009. This GI of ~28-kb has been given the designation GI*Vch*S12 and contains an integrase with 94% nucleotide and 100% amino acid identity to the VPI-1 integrase gene and protein, respectively, and characteristic VPI-1 *attL* and *attR* sites abutting the GI^44^. Given GI*Vch*S12’s location, with identical integrase protein and *att* sites, this GI is likely to have recombination functions identical to VPI-1. Previous studies have observed variations in VPI-1 through PCR analysis of *V. cholerae* strains or BLASTN analysis of *V. cholerae* genomes for VPI-1 genes. For the most part, the variations identified represent minor gene gain/loss events or sequence changes to known ORFs in VPI-1^10^,^11^,^12^. GI*Vch*S12 is different in that it shares practically no gene content with VPI-1.

### A CRISPR-Cas module for self-preservation

Bioinformatic analysis of GI*Vch*S12 identified a CRISPR-Cas module and a T6SS auxiliary locus at the *attR* and *attL* ends, respectively (Fig. 1). The CRISPR-Cas module in GI*Vch*S12 contains genes encoding homologues of Cas1, Cas3, and Cas6. Based on the protein sequences and their organization, this CRISPR-Cas system is most likely similar to those of type 1-F^26^. Several spacers displayed 100% identity to various bacteriophage genomes consistent with the module having a role in acting against invading foreign DNA. The association of a CRISPR-Cas module within a GI is intriguing for two reasons. From an ecological perspective, the mobilization of a CRISPR-Cas system benefits the host not only with an adaptive immune system but also by the instant addition of the immunity that comes with the various spacer sequences it already carries. Thus, a host would immediately gain protection from various bacteriophages and other invading foreign DNA within that ecosystem. Secondly, an intriguing study identified a CRISPR-Cas system within a bacteriophage genome that was able to counteract an inhibitory GI present in the bacterial host genome, thus improving the bacteriophages’ capacity to successfully infect the bacterial host^45^. As a result, this raises the possibility that the CRISPR-Cas system within GI*Vch*S12 might improve integration efficiency in recipient cells that contain other genetic elements interfering with the GI’s integrity and/or integration. Furthermore, once integrated into the host, the CRISPR-Cas system could prevent the replacement of GI*Vch*S12 by VPI-1 or other GIs competing for the same integration sites. The CRISPR-Cas system found on GI*Vch*S12 could therefore promote direct self-preservation or self-preservation by protecting its host.

### A novel T6SS auxiliary locus

Also present on GI*Vch*S12 are genes normally associated with three T6SS loci found in all *V. cholerae* genomes, known as the main locus and auxiliary loci 1 and 2. The GI*Vch*S12 locus structurally resembles the two T6SS auxiliary loci, with the presence of an *hcp* gene, a copy of *vgrG,* a gene encoding a protein with a DUF4123 domain, and putative cargo effectors and immunity proteins further downstream. The lack of proteins making up the T6SS machinery indicates that the proteins on this additional auxiliary locus are dependent on the structural proteins encoded on the main chromosomal T6SS locus for effective translocation into target cells that in S12 is present on contig 00022. The auxiliary loci 1 and 2 are present on contigs 00011 and 00021, respectively, although in contig 00021 the sequence breaks before *hcp*, presumably due to the difficulty of assembling repeat regions. At the end of contig 00011, the first 392-bp of *hcp* from auxiliary locus 1 is present before the sequence breaks. In the small contigs of 00079 and 00082 are the first 275-bp and last 108-bp of an *hcp* homologue, respectively, with *hcp* from contig 00079 presumably from auxiliary locus 2. The auxiliary 1 and 2 *hcp* genes in S12 share 99% nucleotide identity with those in *V. cholerae* N16961 and V52. The *hcp* from GI*Vch*S12 is clearly different to both the auxiliary 1 and 2 *hcp* loci sharing 88% nucleotide identity.

The VgrG protein encoded in GI*Vch*S12 is dissimilar to the chromosomal VgrG proteins but, like VgrG-2, lacks a C-terminal effector domain^46^. The DUF4123 domain found in the protein encoded downstream of the *vgrG* gene indicates a function as an accessory loading proteins like *tap*-1 (VC1417 in the auxiliary 1 locus of *V. cholerae* V52), which is responsible for the loading of cargo effectors with antibacterial activity onto VgrG proteins^47^,^48^. Due to the structural similarity of this locus with chromosomal auxiliary loci, it is likely that the hypothetical protein encoded by ORF 11 (Fig. 1) represents such a cargo effector. Antibacterial T6SS effectors are always accompanied by immunity proteins that provide protection against self-intoxication, making it likely that the homologous proteins encoded by ORFs 12, 14a and 14b act as such. ORF 12 is 61% and 62% identical at the nucleotide level to ORFs 14a and 14b, respectively. ORFs 14a and 14b share 89% nucleotide identity. Expression of effectors from the GI*Vch*S12 T6SS locus is likely to increase the range of T6SS-mediated microbial toxicity as evidenced by the divergent VgrG protein and one or more putative other effectors encoded on GI*Vch*S12. It is therefore likely that this increase in effector repertoire gives cells harboring GI*Vch*S12 an advantage over other cells in T6SS-mediated competition. A series of other genes encoding hypothetical proteins are in close proximity and may have a role in either forming the T6SS apparatus or act as effector proteins. For example, ORFs 13 and 15 (also homologues of each other) encode Zn-binding Pro-Ala-Ala-Arg (PAAR) proteins. Zn-binding PAAR proteins form a conical extension on the VgrG tip and also function to attach effector proteins to the spike^24^. More research is required to gain insight on how *V. cholerae* S12 regulates expression of the different Hcp and effector proteins and to determine the enzymatic activity of such effector proteins.

### A successful and globally distributed genomic island

**Figure 2:**
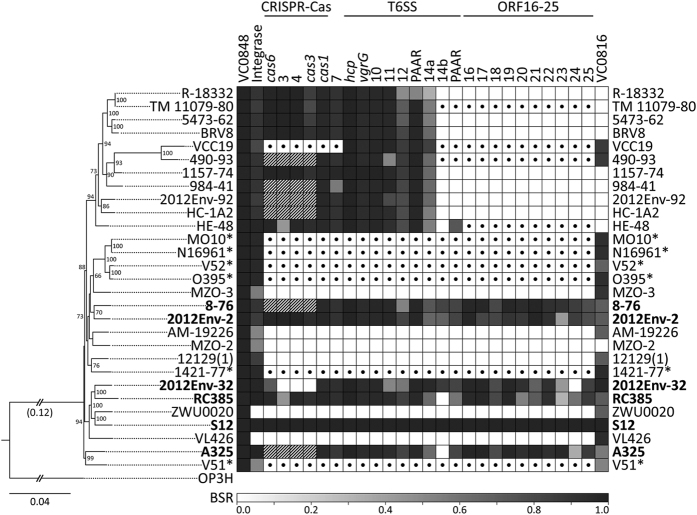
Presence/absence of the GI*Vch*S12 ORFs in various *Vibrio cholerae* strains. The heat map shows the BLAST score ratio (BSR) against the GI*Vch*S12 reference (each column is an ORF). The gradient bar shows the BSR values and their corresponding colours; white indicates the absence of the ORF. Only BSR values of at least 0.3 were included. Strains with similar or complete GI*Vch*S12 are indicated in bold. Striped boxes indicate the presence of a CRISPR-Cas module different from GI*Vch*S12; dotted boxes indicate the presence of other ORFs in those regions different from GI*Vch*S12. Strains with the VPI-1 similar to N16961 are indicated with *. The phylogenetic relationship of the *V. cholerae* strains is shown on the left of the heat map with *Vibrio metoecus* OP3H as outgroup. The maximum likelihood phylogenetic tree was constructed from the core alignment of whole genomes (≈2.5 mb) and using the GTR gamma substitution model with 100 bootstrap replicates (indicated on the tree nodes). The scale bar represents nucleotide substitutions per site. Shortened branch lengths, approximately three times the scale bar (0.12), are indicated.

In order to determine whether GIs similar to GI*Vch*S12 were present in the VPI-1 site of other *V. cholerae* genomes, we compared GI*Vch*S12 to 28 other *V. cholerae* strains for which genome sequences are available in public databases. The non-O1/O139 strain from Haiti (2012Env-2) contained a complete GI*Vch*S12-like island with similar CRISPR-Cas and T6SS modules and a complete set of ORFs 16–25 (Fig. 2). RC385 has an almost complete GI*Vch*S12 but lacks ORF14b. A further three GI*Vch*S12-like islands had minor variations. The non-O1/O139 strain 2012Env-32 from Haiti lacks ORF24 and three other ORFs in the CRISPR-Cas module and the non-O1/O139 strain 8–76 and O1 strain A325 from India and Argentina vary in their CRISPR-Cas modules. A325 also lacks ORF14b. Fifteen other GIs were identified containing a CRISPR-Cas module and/or a T6SS, although for many (in the upper most clade; see Fig. 2), we were unable to locate a contig containing VC0816 so it is unclear if the GI continues beyond ORF14a. One GI (in strain VCC19) harboured a T6SS but contained other genes instead of the CRISPR-Cas module and ORFs 16–25. Two other GIs in strains 490–93 and HE-48 contained a divergent CRISPR-Cas modules and other genes instead of ORFs16-25. Given that all these strains have been isolated from different geographic locations (Supplementary Material) including Europe, South America, North America, and Asia, this data indicates that GIs with VPI-1 recombination characteristics are active in disseminating CRISPR-Cas and T6SS modules across the globe.

GI*Vch*S12 and associated islands can be divided into sub-clusters or islets, as has been previously done for *V. cholerae* pathogenicity island 2 (VPI-2)^12^, consisting of the CRISPR-Cas cluster, the T6SS cluster, and the ORFs 16–25 cluster. Given that multiple GIs (Fig. 2) contain different genes in the ORF 16–25 region and in the case of VCC19, in the CRISPR-Cas region, other islets have clearly been acquired by relatives of GI*Vch*S12. We hypothesize that evolution of GI*Vch*S12 and its relatives proceeded through acquisition of these sub-clusters through homologous recombination processes as supported by the observation that the first 293 bp of the GI*Vch*S12 integrase shares 100% identity to the VPI-1 integrase before dropping to 90% for the remainder of the gene.

### A genomic island that readily excises as a circle

**Figure 3:**
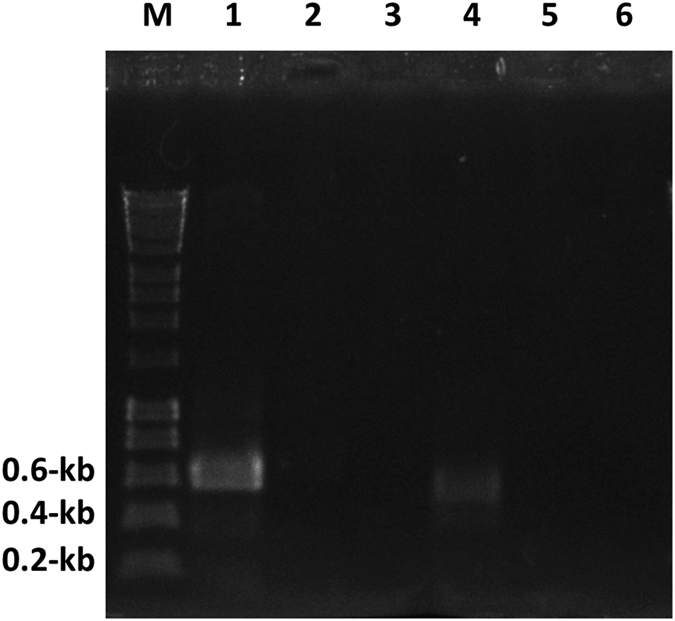
Cropped DNA agarose gel showing the amplicon of a two-stage nested inverse PCR of the excised GI*Vch*S12 circle (lane 1) and the amplicon of a two-stage PCR of the “empty” chromosomal GI*Vch*S12 site (lane 4). Controls for each PCR are given in the adjacent lanes that include nested PCR of the negative control from the first-stage PCR and dH_2_O negative control (lanes 2 and 3 for GI*Vch*S12 circle PCR and lanes 5 and 6 for “empty” chromosomal GI*Vch*S12 site PCR) respectively. Lane M is the DNA marker with relevant band sizes shown on the left of the gel.

To confirm that the GI could excise as a circle, a nested PCR strategy was employed using primers annealing within the GI and facing outward toward the *attL* and *attR* ends. First, primers ML130 and ML131 were used in a PCR reaction with template derived from a plasmid preparation of *V. cholerae* S12. Next, 1 μl from the ML130/ML131 PCR was used as template for a fresh PCR reaction employing primers ML126 and ML127 (relative primer binding sites are shown in Fig. 1A) giving an expected fragment of ~580-bp (Fig. 3). As expected, sequence of the PCR product showed that excision occurs at the *att* sites abutting the GI producing an *attP* site identical to what is observed when VPI-1 excises from the genome^44^.

PCR of the empty chromosomal site was also conducted using a nested PCR strategy with primers annealing outside the GI and facing in toward the *attL* and *attR* ends. Primers ML134 and ML135 were used in a PCR reaction with template derived from a PCR reaction with ML136 and ML137 using genomic DNA as template. The resulting product (Fig. 3) was sequenced and showed precise excision of GI*Vch*S12 leaving an identical *attB* site as previously seen with excision of VPI-1^44^. VPI-1 uses both a phage-like integrase and a transposase protein called VpiT to facilitate excision of the GI^44^. Genes encoding these proteins are present within VPI-1.

However, in some *V. cholerae* pandemic strains, *vpiT* is in a different location^44^. VpiT or a homologue of VpiT was not found in GI*Vch*S12 or in the S12 genome.

In conclusion, we report an interesting GI with VPI-1 recombination characteristics housing a CRISPR-Cas element and a T6SS auxiliary locus. This GI is likely to provide its host with a competitive advantage by protecting from bacteriophages as well as adding T6SS-associated bactericidal effectors proteins. Furthermore, this study shows that GIs with VPI-1 recombination characteristics carrying CRISPR-Cas and T6SS modules are circulating in natural *V. cholerae* populations globally.

## Additional Information

**How to cite this article**: Labbate, M. *et al.* A genomic island in *Vibrio cholerae* with VPI-1 site-specific recombination characteristics contains CRISPR-Cas and type VI secretion modules. *Sci. Rep.* **6**, 36891; doi: 10.1038/srep36891 (2016).

**Publisher’s note:** Springer Nature remains neutral with regard to jurisdictional claims in published maps and institutional affiliations.

## Supplementary Material

Supplementary Information

## Figures and Tables

**Table 1:** Primers used in this study.

Primer	Sequence (5′-3′)	Target
ML126	ACTTCTCGAAAGCGGATCAA	*attL* end of GI*Vch*S12
ML127	AAGCCATCACCATCGAAAAG	*attR* end of GI*Vch*S12
ML130	GCTACCTTTGGCTTCAATCG	*attR* end of GI*Vch*S12
ML131	TGGCAACAAGATGACTTTATCG	*attL* end of GI*Vch*S12
ML134	TCCCTAGCTTCCGCTTGTAA	Between VC0848 and *attR* of GI*Vch*S12
ML135	TCAGTGATGCAGGTTGTTCA	Within VC0816
ML136	GGGAATTTTGCAGTCTGAGG	Between VC0848 and *attR* of GI*Vch*S12
ML137	ATAGGGAGTGGGGCGTTAAT	Within VC0816
Gap_F1	GCGTTTTTATCAATGGCAAACC	Within ORF 13 of GI*Vch*S12
Gap_R1	ACACAGGGCTACCTCTAGATGG	Within and just past ORF 15 of GI*Vch*S12

## References

[b1] Kaper J., Morris J. Jr & Levine M. Cholera. Clin Microbiol Rev 8, 48–86 (1995). 7704895 10.1128/cmr.8.1.48 PMC172849

[b2] Nelson E. J., Harris J. B., Glenn Morris J., Calderwood S. B. & Camilli A. Cholera transmission: the host, pathogen and bacteriophage dynamic. Nat Rev Micro 7, 693–702 (2009). 10.1038/nrmicro2204 PMC3842031 19756008

[b3] Faruque S. M. & Mekalanos J. J. Pathogenicity islands and phages in *Vibrio cholerae* evolution. Trends in Microbiol 11, 505–510 (2003). 10.1016/j.tim.2003.09.003 14607067

[b4] Dobrindt U., Hochhut B., Hentschel U. & Hacker J. Genomic islands in pathogenic and environmental microorganisms. Nat Rev Microbiol 2, 414–424 (2004). 15100694 10.1038/nrmicro884

[b5] Chun J. *et al.* Comparative genomics reveals mechanisms for short-term and long-term clonal transitions in pandemic *Vibrio cholerae*. Proc Natl Acad Sci USA 106, 15442–15447 (2009). 19720995 10.1073/pnas.0907787106 PMC2741270

[b6] Grim C. J. *et al.* Genome sequence of hybrid *Vibrio cholerae* O1 MJ-1236, B-33, and CIRS101 and comparative genomics with *V. cholerae*. J Bacteriol 192, 3524–3533 (2010). 20348258 10.1128/JB.00040-10 PMC2897672

[b7] Dziejman M. *et al.* Comparative genomic analysis of *Vibrio cholerae*: genes that correlate with cholera endemic and pandemic disease. Proc Natl Acad Sci USA 99, 1556–1561 (2002). 11818571 10.1073/pnas.042667999 PMC122229

[b8] Waldor M. K. & Mekalanos J. J. Lysogenic conversion by a filamentous phage encoding cholera toxin. Science 272, 1910–1914 (1996). 8658163 10.1126/science.272.5270.1910

[b9] Karaolis D. K. R. *et al.* A *Vibrio cholerae* pathogenicity island associated with epidemic and pandemic strains. Proc Natl Acad Sci USA 95, 3134–3139 (1998). 9501228 10.1073/pnas.95.6.3134 PMC19707

[b10] Hasan N. A. *et al.* Nontoxigenic *Vibrio cholerae* non-O1/O139 isolate from a case of human gastroenteritis in the U.S. Gulf Coast. J Clin Microbiol 53, 9–13 (2015). 25339398 10.1128/JCM.02187-14 PMC4290964

[b11] Li M., Kotetishvili M., Chen Y. & Sozhamannan S. Comparative genomic analyses of the *Vibrio* Pathogencity Island and cholera toxin prophage regions in nonepidemic serogroup strains of *Vibrio cholerae*. Appl Environ Microbiol 69, 1728–1738 (2003). 12620865 10.1128/AEM.69.3.1728-1738.2003 PMC150053

[b12] Orata F. D. *et al.* The dynamics of genetic interactions between *Vibrio metoecus* and *Vibrio cholerae*, two close relatives co-occurring in the environment. Genome Biol Evol 7, 2941–2954 (2015). 26454015 10.1093/gbe/evv193 PMC4684700

[b13] Herrington D. A. *et al.* Toxin, toxin-coregulated pili, and the *toxR* regulon are essential for *Vibrio cholerae* pathogenesis in humans. J Exp Med 168 (1988). 10.1084/jem.168.4.1487 PMC2189073 2902187

[b14] Taylor R. K., Miller V. L., Furlong D. B. & Mekalanos J. J. Use of *phoA* gene fusions to identify a pilus colonization factor coordinately regulated with cholera toxin. Proc Natl Acad Sci USA 84, 2833–2837 (1987). 2883655 10.1073/pnas.84.9.2833 PMC304754

[b15] Kovach M. E., Shaffer M. D. & Peterson K. M. A putative integrase gene defines the distal end of a large cluster of ToxR-regulated colonization genes in *Vibrio cholerae*. Microbiology 142, 2165–2174 (1996). 8760931 10.1099/13500872-142-8-2165

[b16] Russell A. B., Peterson S. B. & Mougous J. D. Type VI secretion system effectors: poisons with a purpose. Nat Rev Microbiol 12, 137–148 (2014). 24384601 10.1038/nrmicro3185 PMC4256078

[b17] Pukatzki S. *et al.* Identification of a conserved bacteria protein secretion system in *Vibrio cholerae* using the *Dictyostelium* host model system. Proc Natl Acad Sci USA 103, 1528–1533 (2006). 16432199 10.1073/pnas.0510322103 PMC1345711

[b18] Das S. & Chaudhuri K. Identification of a unique IAHP (IcmF associated homologous proteins cluster in *Vibrio cholerae* and other proteobacteria through in silico analysis. In Silico Biol 3, 287–300 (2003). 12954091

[b19] Williams S. G., Varcoe L. T., Attridge S. R. & Manning P. A. *Vibrio cholerae* Hcp, a secreted protein coregulated with HlyA. Infect Immun 64, 283–289 (1996). 8557353 10.1128/iai.64.1.283-289.1996 PMC173757

[b20] Ballister E. R., Lai A. H., Zuckermann R. N., Cheng Y. & Mougous J. D. *In vitro* self-assembly of tailborable nanotubes from a simple protein building block. Proc Natl Acad Sci USA 105, 3733–3738 (2008). 18310321 10.1073/pnas.0712247105 PMC2268831

[b21] Silverman J. M., Brunet Y. R., Cascales E. & Mougous J. D. Structure and regulation of the type VI secretion system. Ann Rev Microbiol 66, 453–472 (2012). 22746332 10.1146/annurev-micro-121809-151619 PMC3595004

[b22] Brooks T. M., Unterweger D., Bachmann V., Kostiuk B. & Pukatzki S. Lytic activity of the *Vibrio cholerae* Type VI secretion toxin VgrG-3 is inhibited by the antitoxin TsaB. J Biol Chem 288, 7618–7625 (2013). 23341465 10.1074/jbc.M112.436725 PMC3597803

[b23] Dong T. G., Ho B. T., Yoder-Himes D. R. & Mekalanos J. J. Identification of T6SS-dependent effector and immunity proteins by Tn-seq in *Vibrio cholerae*. Proc Natl Acad Sci USA 110, 2623–2628 (2013). 23362380 10.1073/pnas.1222783110 PMC3574944

[b24] Shneider M. M. *et al.* PAAR-repeat proteins sharpen and diversify the type VI secretion system spike. Nature 500, 350–353 (2013). 23925114 10.1038/nature12453 PMC3792578

[b25] Croucher N. J. *et al.* Horizontal DNA transfer mechanisms of bacteria as weapons of intragenomic conflict. PLoS Biol 14, e1002394, doi: 10.1371/journal.pbio.1002394 (2016). 26934590 PMC4774983

[b26] Makarova K. S. *et al.* Evolution and classification of the CRISPR–Cas systems. Nat Rev Microbiol 9, 467–477 (2011). 21552286 10.1038/nrmicro2577 PMC3380444

[b27] Islam A. *et al.* Indigenous *Vibrio cholerae* strains from a non-endemic region are pathogenic. Open Biol 3, 120181 (2013). 23407641 10.1098/rsob.120181 PMC3603452

[b28] Schmieder R. & Edwards R. Quality control and preprocessing of metagenomic datasets. Bioinformatics (2011). 10.1093/bioinformatics/btr026 PMC3051327 21278185

[b29] Zerbino D. R. & Birney E. Velvet: algorithms for *de novo* short reads assembly using de Bruijn graphs. Genome Res 18, 821–829 (2008). 18349386 10.1101/gr.074492.107 PMC2336801

[b30] Boetzer M., Henkel C. V., Jansen H. J., Butler D. & Pirovano W. Scaffolding pre-assembled contigs using SSPACE. Bioinformatics 27, 578–579 (2011). 21149342 10.1093/bioinformatics/btq683

[b31] Boetzer M. & Pirovano W. Toward almost closed genomes with GapFiller. Genome Biol (2012). 10.1186/gb-2012-13-6-r56 PMC3446322 22731987

[b32] Darling A. E., Mau B. & Perna N. T. progressiveMAUVE: multiple genome alignment with gene gain, loss and rearrangement. PLoS One 5, e11147 (2010). 20593022 10.1371/journal.pone.0011147 PMC2892488

[b33] Seemann T. Prokka: rapid prokaryotic genome annotation. Bioinformatics 30 2068–2069 (2014). 24642063 10.1093/bioinformatics/btu153

[b34] Altschul S. F., Gish W., Miller W., Myers E. W. & Lipman D. J. Basic local alignment search tool. J Mol Biol 215, 403–410 (1990). 2231712 10.1016/S0022-2836(05)80360-2

[b35] Grissa I., Vergnaud G. & Pourcel C. CRISPRFinder: a web tool to identify clustered regularly interspaced short palindromic repeats. Nucl Acids Res 35, W52–W57 (2007). 17537822 10.1093/nar/gkm360 PMC1933234

[b36] Aziz R. K. *et al.* The RAST server: rapid annotation using subsystems technology. BMC Genomics 9, 75 (2008). 18261238 10.1186/1471-2164-9-75 PMC2265698

[b37] Rasko D. A., Myers G. S. A. & Ravel J. Visualization of comparative genomic analyses by BLAST score ratio. BMC Bioinformatics 6, 1–7, doi: 10.1186/1471-2105-6-2 (2005). 15634352 PMC545078

[b38] Rost B. Twilight zone of protein sequence alignments. Protein Engineering 12, 85–94 (1999). 10195279 10.1093/protein/12.2.85

[b39] Angiuoli S. V. & Salzberg S. L. Mugsy: Fast multiple alignment of closely related whole genomes. Bioinformatics 27, 334–342 (2010). 21148543 10.1093/bioinformatics/btq665 PMC3031037

[b40] Goecks J., Nekrutenko A. & Taylor J. Galaxy: a comprehensive approach for supporting accessible, reproducible, and transparent computational research in the life sciences. Genome Biology 11, 1–13 (2010). 10.1186/gb-2010-11-8-r86 PMC2945788 20738864

[b41] Kearse M. *et al.* Geneious Basic: An integrated and extendable desktop software platform for the organization and analysis of sequence data. Bioinformatics 28, 1647–1649 (2012). 22543367 10.1093/bioinformatics/bts199 PMC3371832

[b42] Stamatakis A. RAxML version 8: a tool for phylogenetic analysis and post-analysis of large phylogenies. Bioinformatics 30, 1312–1313 (2014). 24451623 10.1093/bioinformatics/btu033 PMC3998144

[b43] Kirchberger P. C. *et al.* *Vibrio metoecus* sp. nov., a close relative of *Vibrio cholerae* isolated from coastal brackish ponds and clinical specimens. Int J Syst Evol Microbiol 64, 3208–3214 (2014). 24972615 10.1099/ijs.0.060145-0

[b44] Rajanna C. *et al.* The *Vibrio* pathogenicity island of epidemic *Vibrio cholerae* forms precise extrachromosomal circular excision products. J Bacteriol 185, 6893–6901 (2003). 14617653 10.1128/JB.185.23.6893-6901.2003 PMC262723

[b45] Seed K. D., Lazinski D. W., Calderwood S. B. & Camilli A. A bacteriophage encodes its own CRISPR/Cas adaptive response to evade host innate immunity. Nature 494, 489–491 (2013). 23446421 10.1038/nature11927 PMC3587790

[b46] Pukatzki S., Ma A. T., Revel A. T., Sturtevant D. & Mekalanos J. J. Type VI secretion system translocates a phage tail spike-like protein into target cells where it cross-links actin. Proc Natl Acad Sci USA 104, 15508–15513 (2007). 17873062 10.1073/pnas.0706532104 PMC2000545

[b47] Unterweger D. *et al.* Chimeric adaptor proteins translocate diverse type VI secretion system effectors in *Vibrio cholerae*. EMBO 34, 2198–2210 (2015). 10.15252/embj.201591163 PMC4557670 26194724

[b48] Liang X. *et al.* Identification of divergent type VI secretion effectors using a conserved chaperone domain. Proc Natl Acad Sci USA 112, 9106–9111 (2015). 26150500 10.1073/pnas.1505317112 PMC4517263

