# Supplementary material for: A genomic island in Vibrio cholerae with VPI-1 site-specific recombination characteristics contains CRISPR-Cas and type VI secretion modules
Source: Sci Rep. 2016 Nov 15;6:36891. doi: 10.1038/srep36891 (PMC5109276; doi:10.1038/srep36891)
Supplement: Supplementary Information [file srep36891-s1.pdf]

A genomic island in *Vibrio cholerae* with VPI-1 site-specific recombination characteristics contains CRISPR-Cas and type VI secretion modules

Maurizio Labbate, Fabini D. Orata, Nicola K. Petty, Nathasha D. Jayatilleke, William L. King, Paul C. Kirchberger, Chris Allen, Gulay Mann, Ankur Mutreja, Nicholas R. Thomson, Yan Boucher, Ian G. Charles

Table S1: List of whole genome sequences used in this study.

Species	Strain	Serogroup/Serotype	Biotype	Geographic Origin	Source	Year	Accession Number
Vibrio cholerae	**S12**	**Non-O1/O139**		**Sydney, Australia**	**Environmental**	**2009**	**MDST00000000**
Vibrio cholerae	1157-74	O53		India	Clinical	1974	JIDL00000000
Vibrio cholerae	12129(1)	O1	El Tor	Australia	Environmental	1985	ACFQ00000000
Vibrio cholerae	1421-77	O80		India	Clinical	1977	JMBL00000000
Vibrio cholerae	2012Env-2	Non-O1/O139		Lassalle, Haiti	Environmental	2012	JSTD00000000
Vibrio cholerae	2012Env-32	Non-O1/O139		Four-au-chaux, Haiti	Environmental	2012	JSTF00000000
Vibrio cholerae	2012Env-92	Non-O1/O139		Gressier, Haiti	Environmental	2012	JSTJ00000000
Vibrio cholerae	490-93	O155		Thailand	Clinical	1993	JIDQ00000000
Vibrio cholerae	5473-62	O31		Philippines	Clinical	1962	JIDI00000000
Vibrio cholerae	8-76	O77		India	Clinical	1976	JIDN00000000
Vibrio cholerae	984-81	O89		India	Clinical	1981	JMBM00000000
Vibrio cholerae	A325	O1	Inaba	Argentina		1993	CWSO00000000
Vibrio cholerae	AM-19226	O39		Bangladesh	Clinical	2001	AATY00000000
Vibrio cholerae	BRV8			United Kingdom	Clinical		CTBD00000000
Vibrio cholerae	HC-1A2			Haiti	Clinical	2010	AJRO00000000
Vibrio cholerae	HE-48	Non-O1/O139		Haiti	Environmental	2010	AFOR00000000
Vibrio cholerae	MO10	O139		Madras, India	Clinical	1992	AAKF00000000
Vibrio cholerae	MZO-2	O14		Bangladesh	Clinical	2001	AAWF00000000
Vibrio cholerae	MZO-3	O37		Bangladesh	Clinical	2001	AAUU00000000
Vibrio cholerae	N16961	O1	El Tor	Bangladesh	Clinical	1971	AE003852; AE003853
Vibrio cholerae	O395	O1	Classical	India	Clinical	1965	CP001235; CP001236
Vibrio cholerae	R-18832	O1	Amazonia	Amazonas, Brazil	Clinical	1992	AFSV00000000
Vibrio cholerae	RC385	O135		Chesapeake Bay, USA	Environmental	1998	AAKH00000000
Vibrio cholerae	TM 11079-80	O1	El Tor	Brazil	Environmental	1980	ACHW00000000
Vibrio cholerae	V51	O141		USA	Clinical	1987	AAKI00000000
Vibrio cholerae	V52	O37		Sudan	Clinical	1968	AAKJ00000000
Vibrio cholerae	VCC19			São Paulo, Brazil	Environmental	1994	ATEV00000000
Vibrio cholerae	VL426	Non-O1/O139	Albensis	Maidstone, Kent, UK	Environmental		ACHV00000000
Vibrio cholerae	ZWU0020			Missouri, USA	Environmental		JRJX00000000
Vibrio metoecus	OP3H			Oyster Pond, USA	Environmental	2006	JJMN00000000
